# Obesity and Voiding Parameters in a Community-Based Population of Okinawa, Japan: Kumejima Digital Health Project (KDHP)

**DOI:** 10.3390/metabo12050468

**Published:** 2022-05-23

**Authors:** Asuka Ashikari, Minoru Miyazato, Koshi Nakamura, Kiyoto Yamashiro, Takehiro Nakamura, Tsugumi Uema, Moriyuki Uehara, Hiroaki Masuzaki, Seiichi Saito, Shiro Maeda, Hajime Ishida, Masayuki Matsushita

**Affiliations:** 1 Department of Urology, Graduate School of Medicine, University of the Ryukyus, Nishihara 903-0215, Japan; ssaito@med.u-ryukyu.ac.jp; 2 Department of Systems Physiology, Graduate School of Medicine, University of the Ryukyus, Nishihara 903-0215, Japan; miyaz929@med.u-ryukyu.ac.jp; 3 Department of Public Health and Hygiene, Graduate School of Medicine, University of the Ryukyus, Nishihara 903-0215, Japan; knakamur@med.u-ryukyu.ac.jp; 4 Division of Endocrinology, Diabetes and Metabolism, Hematology, Rheumatology (Second Department of Internal Medicine), Graduate School of Medicine, University of the Ryukyus, Nishihara 903-0215, Japan; y.kiyoto419psfm@gmail.com (K.Y.); maystorm180@hotmail.com (T.N.); ma2.ky8.26@gmail.com (T.U.); dairiman@hotmail.co.jp (M.U.); hiroaki@med.u-ryukyu.ac.jp (H.M.); 5 Department of Advanced Genomic and Laboratory Medicine, Graduate School of Medicine, University of the Ryukyus, Nishihara 903-0215, Japan; smaeda@med.u-ryukyu.ac.jp; 6 Division of Clinical Laboratory and Blood Transfusion, University of the Ryukyus Hospital, Nishihara 903-0215, Japan; 7 Department of Human Biology and Anatomy, Graduate School of Medicine, University of the Ryukyus, Nishihara 903-0215, Japan; ishidaha@med.u-ryukyu.ac.jp; 8 Department of Molecular and Cellular Physiology, Graduate School of Medicine, University of the Ryukyus, Nishihara 903-0215, Japan; masayuki@med.u-ryukyu.ac.jp

**Keywords:** obesity, nocturia, polyuria, hours of undisturbed sleep, maximum bladder capacity

## Abstract

(1) Background: Evidence has accumulated regarding the etiology of lower urinary tract symptoms associated with obesity and metabolic syndrome. Therefore, the present study aimed to identify which subjectively and objectively measured voiding parameters were associated with obesity in a community-based population. (2) Methods: Voiding parameters on a self-administered questionnaire and a digital self-health monitoring system for urine excretion (s-HMSU) were compared between participants with and without obesity, defined as a body mass index ≥ 25 kg/m^2^ (*n* = 30 and 29, respectively), from a community in Okinawa, Japan. Logistic regression analysis was employed to calculate the odds ratios of abnormalities in voiding parameters for the obese group, with the non-obese group serving as a reference. (3) Results: The obese group had odds ratios of 5.17 (95% confidence interval: 1.33–20.0) for shortened hours of undisturbed sleep (<302 min) by s-HMSU and 7.65 (1.88–31.1) for nighttime urinary frequency by a questionnaire after adjusting for age and sex. In addition, the obese group had an adjusted odds ratio of 2.27 (0.76–6.78) for decreased maximum bladder capacity (<212 mL) by s-HMSU. (4) Conclusion: the results of the present study suggest that nocturia and shortened hours of undisturbed sleep are signs of obesity.

## 1. Introduction

Lower urinary tract symptoms (LUTS) are a major problem in middle-aged and older populations. Nocturia worsens the quality of daily living because of insomnia and also increases the risk of falls/bone fracture, frailty, pneumonia, and cardiovascular disease [1,2,3]. There have been numerous reports about the association between nocturia and obesity/metabolic syndrome (MetS) based on large-scale, community-based, epidemiological studies [4,5,6]. There has also been accumulating evidence regarding how the etiology of LUTS, such as an overactive bladder or benign prostatic hyperplasia, is related to obesity/MetS [4,7,8,9,10]. However, evidence is scarce on how nocturia itself is related to obesity/MetS. In addition, which specific voiding parameters that are associated with obesity/MetS remain unclear.

The etiology of nocturia is multifactorial and now considered to consist of the following three pathological conditions: overproduction of urine (global/nocturnal polyuria), reduction of bladder capacity, and sleep disorders [11]. As the therapeutic approach for nocturia depends on its cause, the frequency volume chart (FVC) is useful for diagnosing distinct etiologies [12]. Using this self-reported FVC, concrete void parameters, such as nighttime urinary frequency, maximum bladder capacity (MBC), and hours of undisturbed sleep (HUS), can be measured. However, paper-based FVC is annoying and time-consuming for patients with nocturia, and is usually difficult to complete. We recently developed the digital self-health monitoring system of urine excretion (s-HMSU) as an alternative to FVC [13,14]. We believe that the s-HMSU’s ability to automatically record each voiding time and measure the volume of voided urine might be useful in the development of digital health approaches. Therefore, in the present study, we aimed to identify which voiding parameters, including self-reported LUTS questionnaire items and s-HMSU-derived voiding parameters, are associated with obesity by comparing these parameters between individuals with and without obesity, recruited as part of the Kumejima Digital Health Project (KDHP).

## 2. Results

The non-obese and obese groups included 29 and 30 participants, respectively. The age and male-to-female ratio showed no statistically significant differences between the two groups (Table 1). Clinical characteristics, including body mass index (BMI), abdominal circumference, blood pressure, and triglyceride levels, were significantly higher in the obese group than in the non-obese group (*p* < 0.05). Table 2 shows the relevant urinary tract symptoms based on a self-administered questionnaire for the non-obese and obese groups. The nighttime urinary frequency (Q2 in the Core Lower Symptoms Score (CLSS)) was significantly higher in the obese group than in the non-obese group (once and zero, respectively; *p* = 0.012), with 51.7% for zero, 34.5% for once, and 6.9% for two or three times in the non-obese group, and 23.3%, 56.7%, and 20.0% in the obese group. Mid-nocturnal insomnia (Q2 in the Quick Inventory Depressive Symptomatology-Japanese (QIDS-J)) was significantly worsened in the obese group than that in the non-obese group, with it defined as either “I wake up more than once a night and stay awake for 20 min or more, frequently more than half a week (score 3)” or “I wake up at least once a night, but I go back to sleep easily (score 2)” (*p* = 0.0058, Table 2). Table 3 shows the voiding parameters measured using the 24 h voided volume monitoring system for each group. Hours of undisturbed sleep (HUS) tended to worsen in the obese group than in the non-obese group, although the difference was not statistically significant. The average urine flow rate (mL/s) was not significantly different between the groups. When LUTS and voiding parameters were treated as dichotomous variables, the obese group had odds ratios of 5.17 (95% confidence interval (CI): 1.33–20.0, *p* = 0.0175) for shortened HUS (<302 min) and 7.65 (95% CI: 1.88–31.1, *p* = 0.0045) for nighttime urinary frequency (≥1 time; Q2 in CLSS) after adjusting for age and sex, with the non-obese group serving as a reference (Table 4). In addition, the obese group had adjusted odds ratios of 2.45 (95% CI: 0.67–9.14, *p* = 0.1724) for nighttime urinary frequency, documented by the 24 h voided volume monitoring system, and 2.27 (95% CI 0.76–6.78, *p* = 0.1780) for decreased MBC (<212 mL).

## 3. Discussion

The results of the current study indicate the following: (1) Self-reported nighttime urinary frequency (Q2 in CLSS) was greater in the obese group than in the non-obese group, while that measured by s-HMSU tended to be higher in the obese group than in the non-obese group. (2) HUS measured by s-HMSU was significantly shorter in the obese group than in the non-obese group. (3) Mid-nocturnal insomnia obtained from the self-reported questionnaire (QIDS-J Q2) was significantly worse in the obese group. These novel findings suggest that nighttime urinary frequency and shorted HUS are closely related to, and may be a notable sign of, obesity.

Of the three major causes of nocturia (nocturnal polyuria, bladder storage dysfunction, and insomnia) [11], nocturnal polyuria accounts for approximately 80% of the causes [11,12]. In the present study, the nocturnal polyuria index (Npi) was broadly similar and not very high in the non-obese and obese groups (24.1% vs. 25.2%). Day–night reversal, such as decreased secretion of arginine vasopressin and increased blood pressure at night, may be present in older patients with nocturnal polyuria [15,16]. Nocturnal polyuria also results from chronic kidney disease, heart failure, hypertension, hyperglycemia, obstructive sleep apnea, and leg edema [10,17,18,19], suggesting a late marker of MetS. In the present study, creatinine levels were broadly normal in both the non-obese and obese groups. Therefore, organ damage, such as renal function, might not have occurred in our participants, which might not have affected nocturnal polyuria in the present study. These results also suggest that nocturia may be an early sign of obesity/Mets.

The HUS is defined as the time from falling asleep to first awakening, which may be another key factor affecting nocturia-related sleep disturbances and quality of life [20]. Taking sufficient time for slow-wave sleep during non-rapid eye movement sleep, which emerges just after falling asleep, leads to not only a high quality of sleep but also to better glucose metabolism, hormone release, immunity, and memory [21]. It is recommended that HUS should be achieved for at least 3 h to ensure good quality of sleep [20,22]. In the present study, the median value of HUS in the non-obese group was >5 h while the corresponding median value in the obese group was <4 h. Over half of the non-obese individuals did not wake up at night. These results suggest a close association between obesity and nocturia/shortened HUS in our participants.

In Japan, MetS is defined as an abdominal circumference ≥85 cm in men and ≥90 cm in women, accompanied by any two or more of the three disorders (high blood glucose, dyslipidemia, and high blood pressure) [23]. In the present study, eight participants and one participant (27% vs. 3%) met the criteria for MetS in the obese and non-obese groups, respectively. Thus, our participants were relatively young and had a relatively low rate of MetS even in the obese group. However, nighttime urinary frequency in CLSS and HUS by s-HMSU was significantly different between the two groups and there was a high OR for obesity in these two parameters. The results of the present observational study indicate that nocturia (≥1 time) and shorter HUS could be clear symptoms of obesity, including MetS.

In the present study, MBC in the obese group tended to be lower than that in the non-obese group. The reduced bladder capacity commonly occurs with aging (i.e., overactive bladder) [24,25]. In a male rat model of obesity/type 2 diabetes, Kending et al. reported that the urine volume per void was decreased and the number of voids was increased in 16-week-old type 2 diabetes rats compared to controls; however, in 27-week-old type 2 diabetes rats, both parameters were increased, accompanied by a change in the response to adenosine triphosphate in the bladder [26]. This indicated changes in bladder function according to the duration of diabetes. Moreover, an animal experimental study reported that atherosclerosis-induced chronic bladder ischemia induced detrusor overactivity by oxidative stress and proinflammatory cytokines in a rat model of iliac arterial endothelial injury [27,28]. Hypertension could also affect the pelvic arterial blood flow, resulting in the loss of the smooth muscle of the bladder with a decrease in bladder compliance [29,30]. Thus, bladder ischemia, derived from pelvic arterial sclerosis, could be a cause of the reduced bladder capacity in the obese participants.

Our KDHP conducted an interventional study using digital health approaches for comprehensive lifestyle modifications among individuals with obesity and/or MetS, which is increasing in Japan [31]. In this previous study, nighttime urinary frequency did not have a significant change between baseline and 6 months in the intervention group, although it significantly worsened at 6 months in the non-intervention group compared to baseline (1.0 ± 0.7 vs. 1.5 ± 0.5, *p* < 0.05) [14]. Therefore, s-HMSU might be beneficial for monitoring nocturia, which could also prevent its worsening. As supported by this relevant intervention study, our study suggests an association between obesity and nocturia in s-HMSU, including nighttime urinary frequency and HUS. Unlike self-reported and paper-based FVC, digital health approaches, such as the use of s-HMSU, could be convenient and useful for the management of nocturia.

This study had several limitations. First, the number of participants in the present study was small, which could have affected a wide range of confidence intervals. Second, the single-day monitoring period of the s-HMSU was insufficient considering that the recommended period for FVC monitoring is ≥ 3 days [13]. Third, only the CLSS questionnaire was employed to evaluate LUTS. Fourth, we had no data on benign prostatic hyperplasia and pelvic organ prolapse, which also causes LUTS in older men and women, respectively. Finally, our study population was comprised of residents of a single remote southern island in Japan. Therefore, caution is needed when generalizing the results of the present study and further studies are necessary to investigate the relationship between obesity and each voiding parameter in a larger general population. Despite these limitations, nocturia and shortened HUS may be signs of obesity.

## 4. Materials and Methods

### 4.1. Study Design and Participants

This observational study was conducted as part of the KDHP between June 2017 and December 2019 [14]. Individuals aged ≥ 20 years were recruited in a community- or hospital-based health checkup between 2017 and 2019 on Kumejima Island, Okinawa, Japan. The total number of individuals ≥ 20 years in Kumejima was 6334 in 2018. The essential inclusion criteria were that participants were able to install the urine monitoring system in the toilet bowl at home and use it for the study period. A total of 62 individuals agreed to participate in this study and were classified into two groups of 32 and 30 individuals with and without obesity, respectively, according to the presence or absence of obesity, defined as BMI ≥ 25 kg/m^2^ [32]. After, 30 and 29 individuals with and without obesity, respectively, completed the survey for the study and were finally included in the subsequent analyses.

### 4.2. Urinary Tract Symptoms Questionnaire

Participants were asked to fill out a questionnaire including the CLSS and the QIDS-J score [33]. Nighttime urinary frequency (Q2 in CLSS), urinary urgency (Q3 in CLSS), and mid-nocturnal insomnia (Q2 in QIDS-J) were used to assess nocturia.

### 4.3. 24 h Voided Volume Monitoring System

To measure the 24 h voided volume, we employed the s-HMSU system (Symax Inc., Tokyo, Japan) [13,14]. Calibration was performed before monitoring according to the shape, volume, and temperature of each toilet bowl. The s-HMSU system includes a measurement sensor, server, and user terminal. The sensor was installed in the toilet bowel and the volume of urine on every voiding occasion was calculated for each participant. Data automatically collected through the s-HMSU included daytime urinary frequency, nighttime urinary frequency, daytime urine volume (mL), nocturnal urine volume (mL), MBC (mL), and HUS (min). HUS was defined as the time from falling asleep to the first awakening to void [34]. NPi (a percentage) was calculated as nocturnal urine volume (mL)/(daytime urine volume [mL] + nocturnal urine volume [mL]) × 100. The s-HMSU also recorded the start and end times of voiding; accordingly, the average urine flow rate was calculated as a voided volume per void (mL)/voiding time (seconds).

### 4.4. Other Data Collection

Other data collected during the health checkup included age, sex, anthropometric indices, blood pressure, and blood chemistry results in the fasting state. Body height and weight were measured and BMI was calculated as weight (kg)/height squared (m^2^). Fasting blood glucose, hemoglobin A1c (HbA1c), serum total cholesterol, low-density lipoprotein-cholesterol (LDL-C), high-density lipoprotein-cholesterol (HDL-C), triglycerides, and creatinine levels were measured using standard methods.

### 4.5. Statistical Analysis

Continuous variables are expressed as mean and standard deviation or median and interquartile range. Basic clinical characteristics and voiding parameters were compared between the obese and non-obese groups using the unpaired *t*-test and Mann–Whitney U test. The chi-square test was used for categorical comparisons. Finally, logistic regression analysis was employed to calculate the odds ratio (95% CI) of each voiding abnormality for obesity, with non-obesity serving as a reference. The model incorporated age and sex as covariates. The cutoff value of NPi in the chi-square test was adopted as 33% because nocturnal polyuria in middle-aged and older people is defined as >33% of NPi [11,25]. In contrast, the median of MBC (212 mL) and HUS (302 min) in all cases were adopted as cutoff values. All statistical analyses were performed using JMP^®^ Pro version 15.0.0 software (SAS Institute Inc., Cary, NC, USA). All probability values were two-tailed and the significance level was set at *p* < 0.05.

## 5. Conclusions

This study demonstrated an association between obesity and nocturia in a community-based population. Nocturia and a reduction in HUS could be signs of obesity.

## Figures and Tables

**Table 1:** Characteristics of study participants grouped according to the absence or presence of obesity.

	Total (*N* = 59)	Non-Obesity (*N* = 29)	Obesity (*N* = 30)	*p*-Value
Age (years)	54.2 ± 12.9	56.1 ± 12.9	52.3 ± 12.7	0.2552
Sex, *n* (%)				0.5221
Female	26 (44.1)	14 (48.3)	12 (40.0)	
Male	33 (55.9)	15 (51.7)	18 (60.0)	
Body mass index (kg/m^2^)	25.5 ± 3.77	22.9 ± 1.88	28.0 ± 3.39	<0.0001
Abdominal circumference (cm)	88.8 ± 10.00	82.3 ± 6.73	95.1 ± 8.4	<0.0001
Systolic blood pressure (mmHg)	135.0 ± 19.8	128.2 ± 19.6	141.8 ± 17.9	0.0079
Diastolic blood pressure (mmHg)	80.2 ± 13.5	73.0 ± 10.8	87.3 ± 12.3	<0.0001
Fasting glucose (mg/dL)	84.5 (80–92)	84 (80–89)	85 (79.5–92.5)	0.5743
HbA1c (%)	5.6 ± 0.35	5.6 ± 0.40	5.6 ± 0.31	0.4177
Triglyceride (mg/dL)	104 (81–153)	101 (68–123.5)	119.5 (86.25–244)	0.0243
Total cholesterol (mg/dL)	200.3 ± 25.8	196.2 ± 28.4	204.3 ± 22.7	0.2334
HDL-C (mg/dL)	59.4 ± 16.3	63.0 ± 17.7	56.0 ± 14.3	0.1013
LDL-C (mg/dL)	111.8 ± 28.6	112.4 ± 25.6	111.2 ± 31.6	0.8678
Creatinine (mg/dL)	0.73 ± 0.17	0.72 ± 0.14	0.73 ± 0.19	0.8624

Unpaired *t*-test, Mann–Whitney U test, or chi-square test was used to compare each characteristic. HbA1c, hemoglobin A1c; HDL-C, high density lipoprotein-cholesterol; and LDL-C, low density lipoprotein-cholesterol.

**Table 2:** Self-reported urinary tract symptoms of participants grouped by obese status.

	Non-Obesity (*N* = 29)	Obesity (*N* = 30)	*p*-Value
Daytime urinary frequency, CLSS Q1	0 (0–1)	0 (0–1)	0.6890
Nighttime urinary frequency, CLSS Q2	0 (0–1)	1 (0.75–1)	0.012
Urinary urgency, CLSS Q3	0 (0–1)	0 (0–1)	0.7072
Urinary urgency incontinence, CLSS Q4	0 (0)	0 (0)	0.7078
Stress urinary incontinence, CLSS Q5	0 (0)	0 (0–0.25)	0.6193
Weak urinary stream, CLSS Q6	0 (0–1)	0 (0–1)	0.9229
Straining, CLSS Q7	0 (0)	0 (0)	0.3713
Feeling of incomplete emptying, CLSS Q8	0 (0–1)	0 (0–1)	0.7612
Bladder pain, CLSS Q9	0 (0)	0 (0)	0.2918
Urethral pain, CLSS Q10	0 (0)	0 (0)	0.1325
Mid-nocturnal insomnia, QIDS-J Q2	2 (1–2)	3 (1–3)	0.0058

Median (interquartile range). The Mann–Whitney U test was used to compare each symptom. CLSS, Core Lower Urinary Tract Symptoms Score; QIDS-J, Quick Inventory of Depressive Symptomatology-Japanese Score; QIDS-J, Quick Inventory Depressive Symptomatology-Japanese. CLSS, Q1: 0, < 7; 1, 8–9 times; 2, 10–14 times; and 3, 15 times or more; Q2: 0, 0 times; 1, 1 time; 2, 2–3 times; and 3, 4 times or more; and Q3–Q10: 0, none; 1, occasionally; 2, sometimes; and 3, always; QIDS-J Q2 (mid-nocturnal insomnia): 0, I do not wake up at night; 1, I have a restless, light sleep with a few brief awakenings each night; 2, I wake up at least once a night, but I go back to sleep easily; and 3, I wake up more than once a night and stay awake for 20 min or more, frequently more than half a week.

**Table 3:** Voiding parameters measured by 24 h voided volume monitoring system of participants grouped by obese status.

	Non-Obesity (*N* = 29)	Obesity (*N* = 30)	*p*-Value
Water intake (mL/day)	1708.3 ± 705.9	1925.5 ± 765.8	0.2624
Daytime urinary frequency (times)	6.0 (5–7)	6.0 (4.75–8)	0.6789
Nighttime urinary frequency (times)	1.0 (0–1)	1.0 (0–2)	0.2414
Daytime urinary volume (mL)	587.3 (408.4–861.5)	535.1 (355.5–662.1)	0.3958
Nocturnal urinary volume (mL)	190.4 (124.1–405.8)	195.6 (93.3–283.7)	0.2955
NPi (%)	24.1 (18.0–38.0)	25.2 (12.9–32.9)	0.6712
MBC (mL)	226.9 (171.6–315.6)	186.1 (128.6–293.6)	0.1541
HUS (min)	330 (198.0–416.0)	211.5 (45.75–411.0)	0.1541
Average urine flow rate (mL/s)	23.6 (19.0–25.4)	18.8 (14.0–25.2)	0.5956

Mean ± standard deviation, median (interquartile range) unpaired *t*-test, or Mann–Whitney U test was used to compare each parameter. Npi, nocturnal polyuria index; MBC, maximum bladder capacity; and HUS, hours of undisturbed sleep.

**Table 4:** Odds ratios of voiding abnormalities for obesity (vs. non-obesity).

	Cases in	Cases in	Odds Ratio (95% Confidence Interval)			
	Non-Obesity	Obesity	for Obesity (vs. Non-Obesity)			
	(*N* = 29)	(*N* = 30)	Crude	*p*-Value	Age and Sex-Adjusted	*p*-Value
Nighttime frequency (≥score 1), CLSS Q2	12	23	4.11 (1.32–12.80)	0.0149	7.65 (1.88–31.14)	0.0045
Mid-nocturnal insomnia (≥score 2), QIDS-J Q2	13	20	1.73 (0.53–5.64)	0.3626	1.82 (0.55–6.08)	0.3301
Nighttime frequency (≥1 time), s-HMSU	18	22	1.68 (0.56–5.07)	0.3565	2.45 (0.67–9.14)	0.1724
Increased Npi (>33%), s-HMSU	11	7	0.50 (0.16–1.54)	0.2269	0.57 (0.17–1.89)	0.3602
Decreased MBC (<212 mL), s-HMSU	12	17	1.85 (0.66–5.21)	0.2422	2.27 (0.76–6.78)	0.1780
Shortened HUS (<302 min), s-HMSU	11	18	2.45 (0.86–7.00)	0.0928	5.17 (1.33–20.03)	0.0175

A logistic regression model was used to calculate an odds ratio of each voiding abnormality for obesity, with non-obesity serving as a reference. CLSS, Core Lower Urinary Tract Symptoms Score; QIDS-J, Quick Inventory Depressive Symptomatology-Japanese; s-HMSU, self-health monitoring system for urine excretion; Npi, nocturnal polyuria index; MBC, maximum bladder capacity; and HUS, hours of undisturbed sleep. CLSS Q2: 0, 0 times; 1, 1 time; 2, 2–3 times; and 3, 4 times or more; QIDS-J Q2: 0, I do not wake up at night; 1, I have a restless, light sleep with a few brief awakenings each night; 2, I wake up at least once a night, but I go back to sleep easily; and 3, I wake up more than once a night and stay awake for 20 min or more, frequently more than half a week.

## Data Availability

The datasets analyzed in the current study are available from the corresponding author upon reasonable request.
